# A vector holographic optical trap

**DOI:** 10.1038/s41598-018-35889-0

**Published:** 2018-11-26

**Authors:** Nkosiphile Bhebhe, Peter A. C. Williams, Carmelo Rosales-Guzmán, Valeria Rodriguez-Fajardo, Andrew Forbes

**Affiliations:** 1 School of Physics, University of the Witwatersrand, Private Bag 3, Wits, 2050 South Africa; 2 Mechanical Engineering, Massachusetts Institute of Technology, 33 Massachusetts Ave, Cambridge, MA 02139 USA

## Abstract

The invention of optical tweezers almost forty years ago has triggered applications spanning multiple disciplines and has also found its way into commercial products. A major breakthrough came with the invention of holographic optical tweezers (HOTs), allowing simultaneous manipulation of many particles, traditionally done with arrays of scalar beams. Here we demonstrate a vector HOT with arrays of digitally controlled Higher-Order Poincaré Sphere (HOPS) beams. We employ a simple set-up using a spatial light modulator and show that each beam in the array can be manipulated independently and set to an arbitrary HOPS state, including replicating traditional scalar beam HOTs. We demonstrate trapping and tweezing with customized arrays of HOPS beams comprising scalar orbital angular momentum and cylindrical vector beams, including radially and azimuthally polarized beams simultaneously in the same trap. Our approach is general enough to be easily extended to arbitrary vector beams, could be implemented with fast refresh rates and will be of interest to the structured light and optical manipulation communities alike.

## Introduction

Since their inception in 1970^1^, optical tweezers have become a versatile non-invasive technique to manipulate micro and nano structures. Key to this is the notion that light carries momenta: linear momentum (known at least as far back as Kepler), spin angular momentum^2,3^ and orbital angular momentum^4^, with a change in momentum resulting in a force. In physics for example, the interaction of such optical forces with atomic ensembles paved the way towards the invention of powerful techniques for laser cooling^5–7^, which in turn allowed the realization of the Bose-Einstein condensate^8,9^. In biology, it provided with a perfect non-invasive tool for the study of single molecules and cells, for example, to facilitate the understanding of all the complex dynamic processes occurring inside a cell or to analyze the mechanical properties of DNA^10–15^. Extensive reviews of all the applications that optical tweezers have facilitated can be found in refs^16–20^ and references therein.

A major breakthrough in optical manipulation techniques came from the field of structured light^21^, especially the use of the Spatial Light Modulators (SLMs)^22,23^ to produce holographic optical traps (HOTs)^24–26^. SLMs are computer controlled devices based on liquid crystal technology that allow, amongst other things, a fast and precise generation and control of almost any beam shape, their digital focusing and propagation, as well as the simultaneous generation and detection of high numbers of individual beams^27,28^ which have been used to control multiple particles in 2D and 3D configurations^29–33^ as well as along exotic trajectories^34–42^.

The aforementioned studies were done with scalar light, while recently it has become topical to explore vector states of light^43,44^. Along this line, the generation methods have evolved from simple interferometric techniques, capable of generating only one vector beam, to more complicated configurations relying on spatial light modulators^43,45–48^. There is interest in using such beams in optical traps due to the enhanced focusing ability of certain vector states of light^49–57^ thereby improving the trapping efficiency^49,58^. For example, Michihata *et al*. pointed out that radially polarized beams enhance the axial trapping efficiency near 2× compared to linearly polarized beams, at the expense of reducing the transverse efficiency by about a half^49^. Other studies have shown that radially and azimuthally polarized beams exhibit higher axial trapping forces on core-shell magnetic microparticles as compared to Gaussian beams^50^. Direct comparison of azimuthally and radially polarized vector beams has shown that the former features a stronger lateral trapping force compared to the latter^51,52^. These topical beams are described by states on a generalized Poincaré Sphere^59,60^, the so-called Higher-Order Poincaré Sphere (HOPS), to account for the total angular momentum of light, spin and angular^59^.

Here we introduce a HOT for arbitrary HOPS beams that allows for multiple HOPS beams to be delivered into a trap in some desired array. Each HOPS beam in the array is independently controlled by an SLM and may be switched from scalar states (on the poles of the HOPS) to cylindrical vector vortex beams (on the equator of the HOPS). By this approach, we are able to tailor on-demand the 3D shape of the optical forces at specific locations inside the optical trap. To show the potential of this technique, we simultaneously compare the trapping strength of cylindrical vector vortex and scalar vortex beams in the same trap. While our vector HOT technique is implemented here with an SLM and delivers arrays of HOPS beams, we emphasize that this may easily be extended to faster refresh rates with digital micro-mirror devices (DMDs)^61^ as well as to arbitrary vector states beyond HOPS beams. Our work introduces a new HOT tool which we believe will be beneficial to structured light and optical manipulation studies alike.

## Generating arrays of Poincaré beams

**Figure 1:**
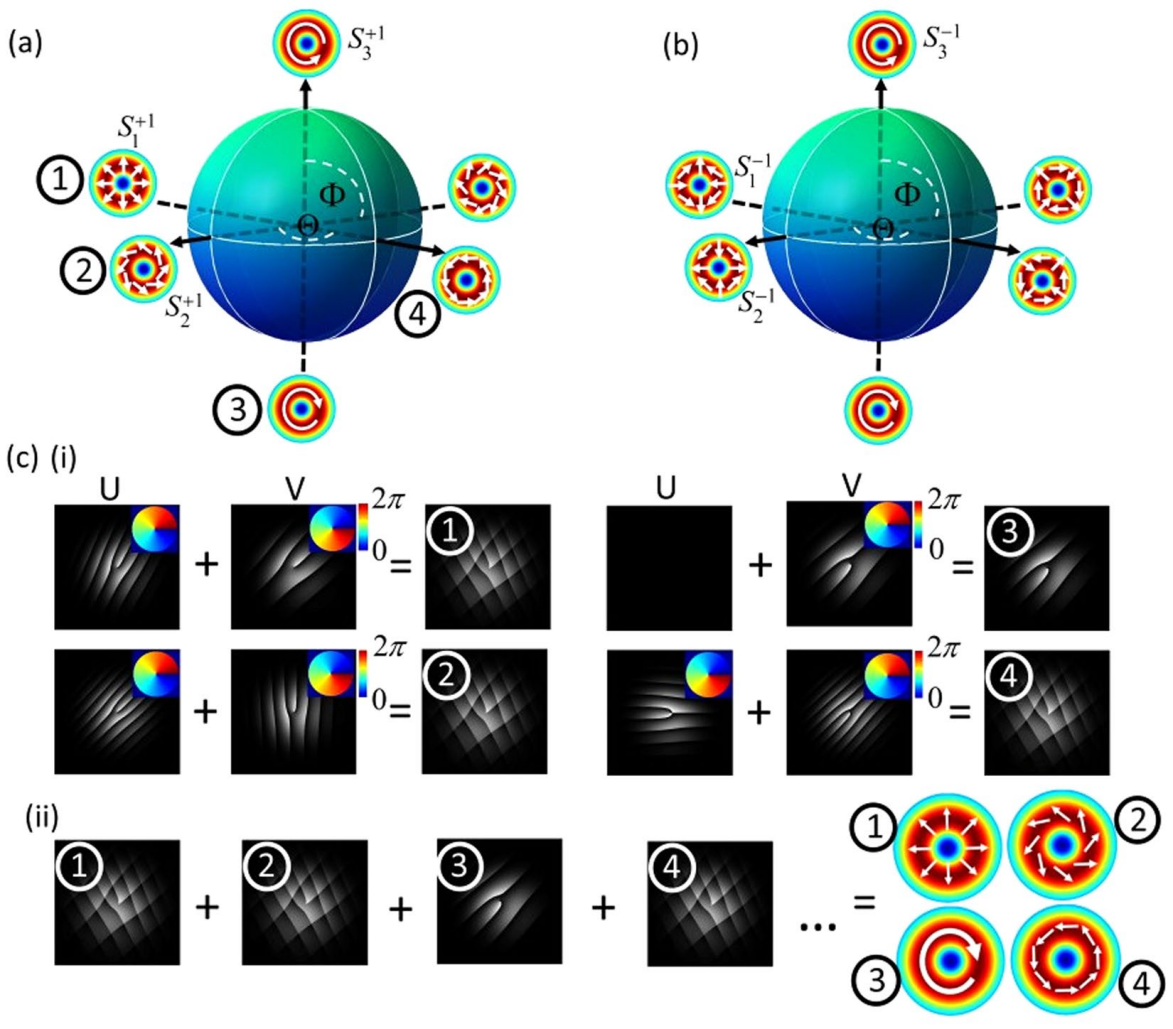
Geometric representation of vector beams with arbitrary polarization states on the higher-order Poincaré sphere, for (**a**) ℓ = l and (**b**) ℓ = −l. Each vector beam is digitally generated by encoding a multiplexed hologram consisting of two beams with unique carrier frequencies, as shown in (**c**) (i), propagating along different paths, that upon coaxial superposition, can create a specific higher-order Poincaré beam (position 1 in (**a**)). Manipulation of the programmed holograms allows digital creation of any arbitrary polarization state on the sphere (examples denoted by positions 2, 3 and 4 in (**a**) and the corresponding hologram pairs are given in (**c**) (i)). Superposition of these multiplexed hologram pairs results in the simultaneous generation of multiple HOPS beams at unique spatial positions as shown in (**c**) (ii).

Vector beams are commonly generated as coaxial superpositions of orthogonal scalar fields with orthogonal polarization states. Mathematically, they can be represented as

1 $$U(r)={U}_{R}\hat{{\bf{R}}}+{U}_{L}\hat{{\bf{L}}},$$

where $\hat{{\bf{R}}}$ and $\hat{{\bf{L}}}$ represent the unitary vectors of the right and left circular polarization, respectively, and *U*_*R*_ and *U*_*L*_ are orthogonal scalar fields. Using the Laguerre Gaussian basis as an example we have

2 $$U(r)=L{G}_{{p}_{1}}^{{\ell }_{1}}{{\rm{e}}}^{i{\alpha }_{1}}\hat{{\bf{R}}}+L{G}_{{p}_{2}}^{{\ell }_{2}}{{\rm{e}}}^{-i{\alpha }_{2}}\hat{{\bf{L}}}.$$

here $L{G}_{{p}_{1}}^{{\ell }_{1}}$ and $L{G}_{{p}_{2}}^{{\ell }_{2}}$ are the amplitudes of each orthogonal component and represent the well-known Laguerre-Gaussian modes with azimuthal and radial indexes ℓ and *p*, respectively, while *α*_1_ and *α*_2_ define modal phases. In this study, we will restrict ourselves to modes with ℓ = −ℓ_2_ = ±ℓ, *α*_1_ = 0 and *α*_2_ = *α* in order to create HOPS beams

3 $$U(r,{\rm{\Phi }},{\rm{\Theta }})=\,\cos (\frac{{\rm{\Phi }}}{2})L{G}_{0}^{\ell }{{\rm{e}}}^{-i{\rm{\Theta }}\mathrm{/2}}\hat{{\bf{R}}}+\,\sin (\frac{{\rm{\Phi }}}{2})L{G}_{0}^{\ell }{{\rm{e}}}^{i{\rm{\Theta }}\mathrm{/2}}\hat{{\bf{L}}},$$

where Φ ∈ [0, *π*] and Θ ∈ [0, 2*π*] are the coordinates on the HOPS, as shown in Fig. 1(a,b). In this geometric representation, the left and right circularly polarized orbital angular momentum (OAM) states are located on the poles (our scalar vortex beams); the cylindrical vector vortex beams lie along the equator; and intermediate states occupy the rest of the sphere. Such beams have been created by a myriad of techniques^47,62^ and found many applications^44,63,64^.

To generate a single vector beam, we first generate two scalar beams by multiplexing their corresponding holograms onto a SLM, each encoded with a unique spatial frequency to ensure their propagation along different paths. This way we can manipulate the polarization on each path independently. To realize HOPS beams is necessary to superimpose appropriate scalar beams with orthogonal polarizations. Since the SLM only modulates one polarization, a HWP was introduced in one path to rotate their polarization. A PBS was used to achieve co-linear recombination followed by a quarter wave plate to create the desired mode. The beauty of this approach lies in the fact that all properties of each individual beam can be manipulated digitally, without any mechanical component, allowing the generation of any beam on the HOPS. For example, to generate all cases shown in Fig. 1(a), labeled as **1**, **2**, **3**, and **4**, one needs only to adjust the relative phase and amplitude of each scalar beam, associated to the parameters Θ and Φ, respectively. For the case of the radially polarized beam, labeled as **1**, the corresponding parameters are: ℓ = 1, Φ = *π*/2 and Θ = 0 (see Fig. 1(c,i) inset **1**). By superimposing each of the multiplexed pairs of holograms into a single hologram, as shown in Fig. 1(c,ii), the simultaneous generation of multiple vector beams can be realized.

## Results

### Arrays of HOPS beams

**Figure 2:**
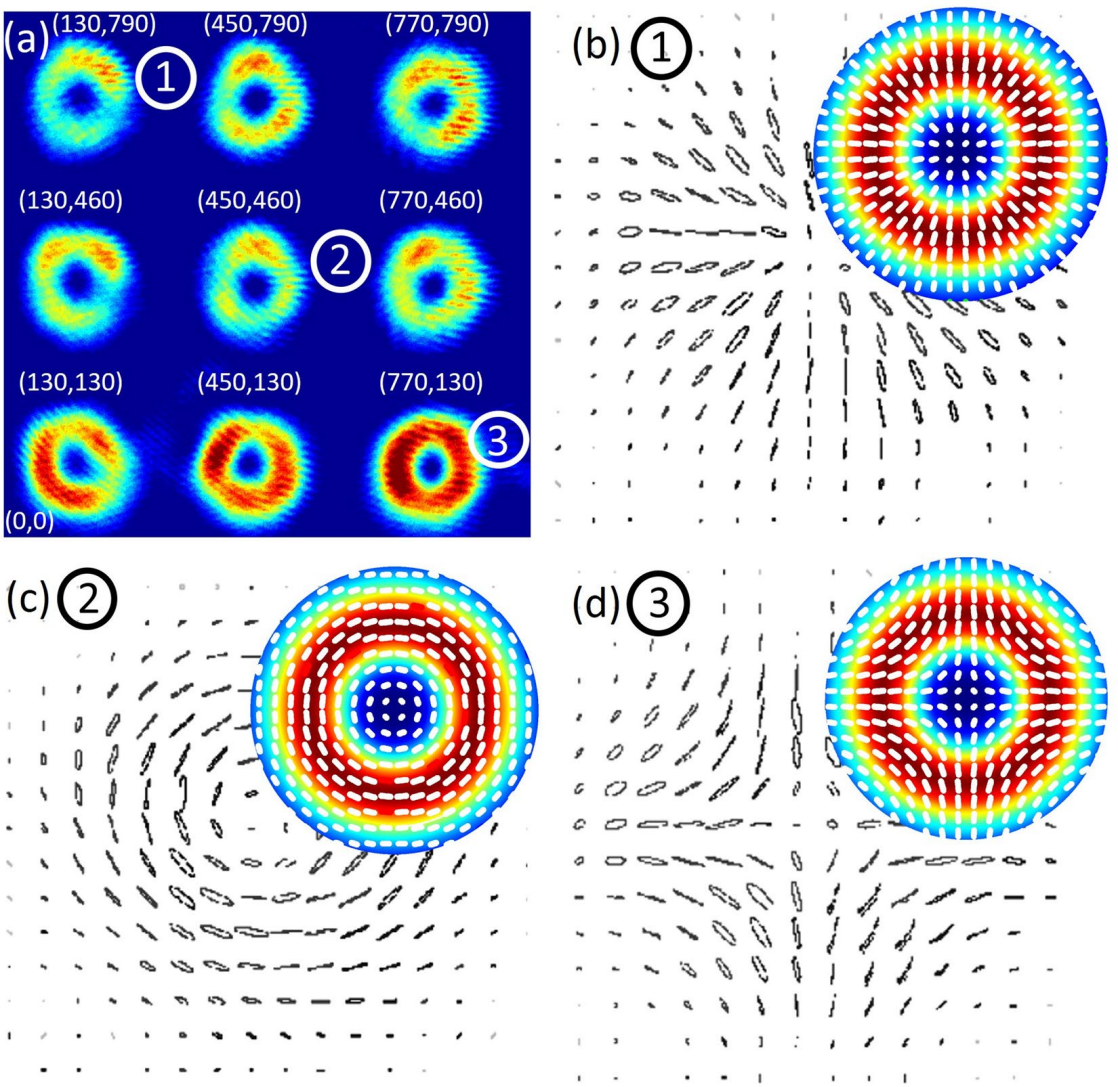
(**a**) Experimental image of a 2D array of 3 × 3 HOPS beams (topological charges of ℓ = 1 and ℓ = 2) at the desired locations in the (*x*, *y*) plane. (**b**–**d**) Shows experimentally determined polarization maps of beams **1**, **2** and **3**, confirming the vector nature of the created beams. The inserts are theoretically simulated intensity and polarization distributions of the beams.

Using our approach, we created a square array of 3 × 3 HOPS beams with topological charges ℓ = 1 and ℓ = 2 for 9 arbitrarily chosen polarization structures, and delivered these through the system. The intensity of the experimentally generated array of HOPS beams is shown in Fig. 2(a). Intensity differences between vector beams in the bottom row in comparison to those at above rows arise from the distinct deflection angles out of the SLM, such that the more intense are less deflected from the undiffracted zero order. Importantly, by adjusting the phase modulation depth on the encoded hologram, equal intensity distributions can be easily attained. All 9 beams were delivered to the desired locations (*x*_*i*_, *y*_*i*_) in the trapping plane, here designed to be in a square array. Table 1 contains the values of the beam positions at the trapping plane as observed on the image, their corresponding grating frequencies (*x*_*u*_, *y*_*u*_) and (*x*_*v*_, *y*_*v*_) for paths **U** and **V**, respectively, and the calculated angles relative to the undiffracted zero order beam as determined according to ref.^46^ These values clearly show that by modifying grating frequencies in the hologram, we can chose the arrangement of the vector beams at will. To determine the quality of each HOPS beam, we performed a spatially resolved stokes measurement^43,65^. Figure 2(b–d) shows the measured polarization distributions of beams **1**, **2** and **3** of the 9 multiplexed vector beams. The inserts show the corresponding theoretically generated intensity and polarization distributions, which are in good agreement with the experimental results confirming the creation of vector beams. Because the beams are created in a 2D array, the measurements could be performed simultaneously on all 9 beams.

**Table 1:** Positions, angles and grating frequencies for the created 3 × 3 square array of HOPS beams.

Position (*x*_*i*_, *y*_*i*_) (pixels)	*θ* (rad)	*x*_*u*_	*y*_*u*_	*x*_*v*_	*y*_*v*_
(130, 790)	0.008	−0.05	0.15	0.21	0.15
(450, 790)	0.008	−0.08	0.15	0.18	0.15
(770, 790)	0.008	−0.11	0.15	0.15	0.15
(130, 460)	0.005	−0.05	0.10	0.21	0.10
(450, 460)	0.005	−0.08	0.10	0.18	0.10
(770, 460)	0.005	−0.11	0.10	0.15	0.10
(130, 130)	0.001	−0.05	0.05	0.21	0.05
(450, 130)	0.001	−0.08	0.05	0.18	0.05
(770, 130)	0.001	−0.11	0.05	0.15	0.05

### 2D Optical traps with arrays of HOPS beams

**Figure 3:**
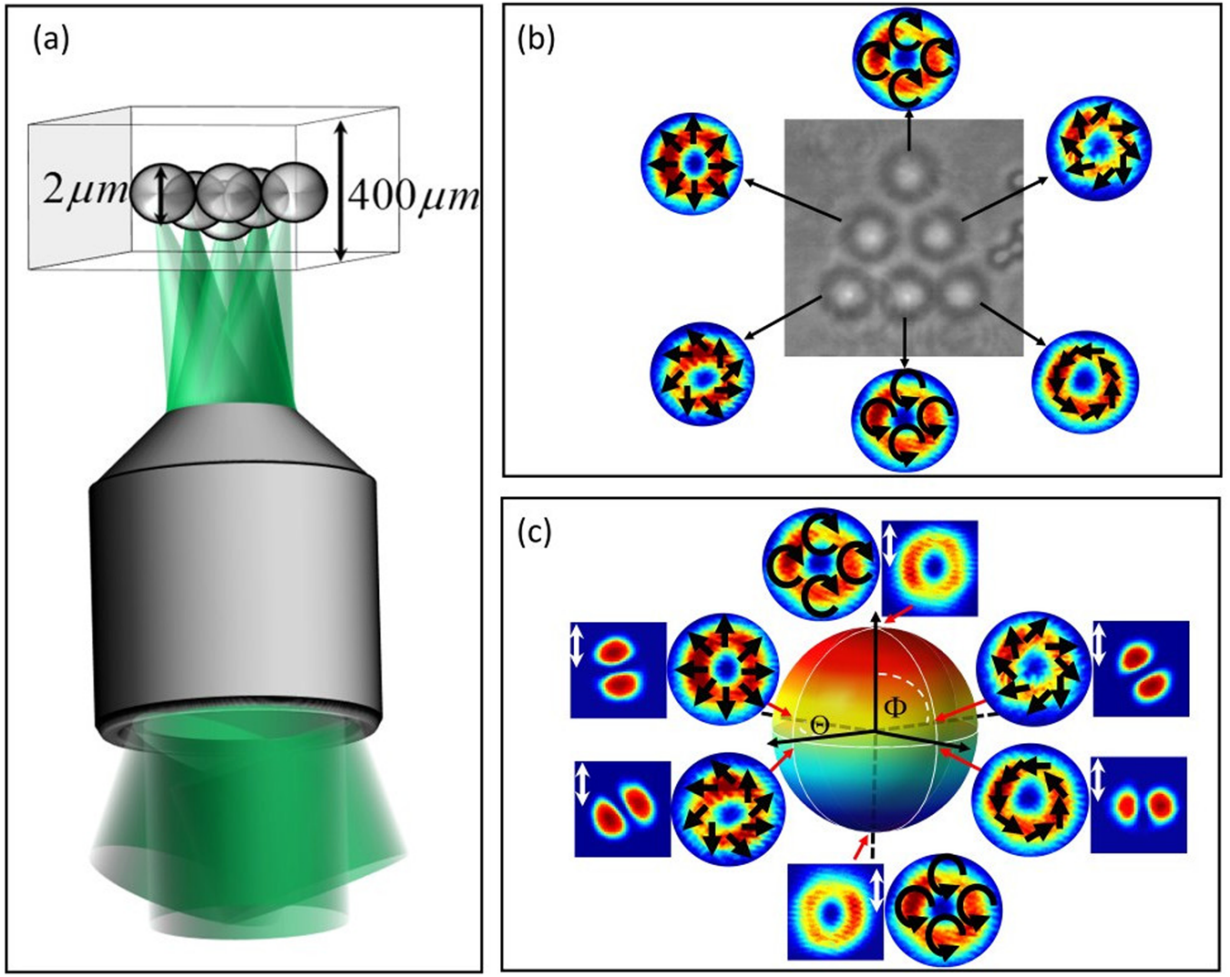
(**a**) Schematic of our vector HOT, with 2 *μ*m silica beads to be trapped in a triangular pattern inside a trapping well of height 400 *μ*m. (**b**) Experimental image of the six trapped beads with the corresponding measured intensity of each trapping HOPS beam, overlaid with the respective polarization structure. The HOPS beams can be geometrically represented on the HOPS as shown in (**c**). Insets in (**c**) show the measured intensity profiles, as well as those after projection through a linear polarizer set to vertical transmission, confirming the state of each mode. Note that here we have demonstrated simultaneous trapping with vector and scalar OAM beams with the same HOT.

In previous sections we described how to create arrays of reconfigurable HOPS beams; now we wish to employ this in a vector HOT. To this end, we projected an array of created vector beams into the entrance pupil of the objective with each HOPS beam at an appropriate incident angle so as to produce the desired 2D array of traps. By way of example, we trapped beads in a triangular array, illustrated in Fig. 3(a) and shown experimentally in (b). Figure 3(b) shows six particles of diameter ≈2 *μ*m, trapped with ≈10 mW of average power arranged in a triangular configuration. Since our approach allows the independent creation of any arbitrary polarization state on the HOPS, we demonstrated multiple optical traps with six arbitrary positions on the HOPS, as shown in Fig. 3(c). Here we also make clear that the vector HOT can also reproduce traditional scalar HOTs: two of the six beams in the triangular array are in fact scalar OAM beams from the poles of the HOPS. All beam insets in Fig. 3(b) are experimentally measured intensity profiles, and insets in (c) show the intensity after a linear polarizer, as explained in the caption.

### Optical forces in our vector HOT

**Figure 4:**
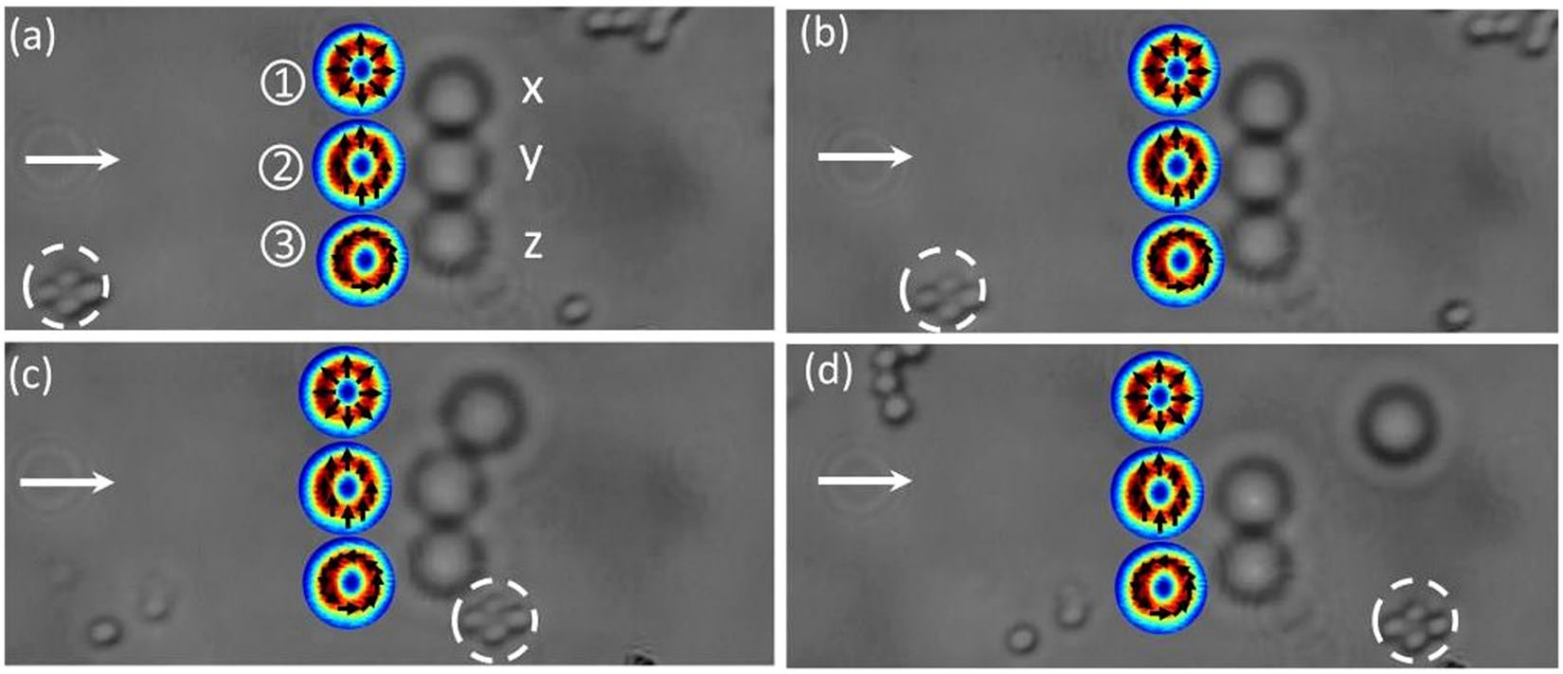
Real-time calibration of multiple vector beam traps consisting of radial, linear and azimuthal polarization states. At some set stage velocity the weakest radially polarized trap (bead 1) results in the bead escaping while the beads in the stronger traps (beads 2 and 3) remain trapped. Arrows show the direction of motion of the stage while the particle in the white dotted circle serves as a stationary reference. See Supplementary Information for the video file.

Finally, as a proof-of-concept application of our technique, we trap three beads simultaneously in a linear array and performed a drag force test. We create HOPS beams that are radially, azimuthally and linearly polarized at the plane of the trap, as shown in Fig. 4. Such a configuration allows for the direct comparison of the trap stiffness for each beam. First, we corroborated that the trapping strength of each beam is position independent. For this, we interchange beam **1** (azimuthal) with beams **2** (linearly) and **3** (azimuthal). That is, from position *x* to position *y* and *z*. We find that the outcome was independent of position. Radially polarized beams exert the weakest trapping force, as compared to azimuthally and linearly polarized beams which remain trapped for higher speeds of ~55 *μ*m/s and 64 *μ*m/s, respectively. Some frames of this test are shown in Fig. 4 and the associated media file.

We measured the lateral trapping strength of each beam, with the results shown in Table 2. From the results we observe that the linearly polarized scalar beam has the strongest transverse trapping force, with the radially polarized beam the least. These results are in agreement with previous reports^49,51,52^.

**Table 2:** Transverse trapping stiffness (*pN*/*μ*m) as a function of laser power (*mW*).

Laserpower (*mW*)	Linear	Azimuthal	Radial	Hybrid odd	Hybrid even
40	2.7 ± 0.4	2.4 ± 0.3	1.8 ± 0.3	2.5 ± 0.2	2.2 ± 0.3
35	2.1 ± 0.3	1.6 ± 0.5	1.4 ± 0.4	1.7 ± 0.5	1.5 ± 0.4
30	1.6 ± 0.2	1.2 ± 0.2	1.1 ± 0.1	1.3 ± 0.2	1.2 ± 0.1
25	1.2 ± 0.2	1.0 ± 0.2	0.9 ± 0.2	1.1 ± 0.1	1.0 ± 0.2
20	0.8 ± 0.2	0.6 ± 0.1	0.5 ± 0.1	0.7 ± 0.1	0.5 ± 0.1
15	0.5 ± 0.03	0.4 ± 0.1	0.4 ± 0.04	0.5 ± 0.05	0.4 ± 0.04

## Discussion

The approach we have outlined allows arbitrary arrays of vector beams to be created and delivered to a trap, resulting in the demonstration of a vector HOT with multiple beams. Importantly, as we have shown, the vector HOT can be reduced to a traditional scalar HOT by simply switching one hologram off, and is versatile enough to allow both scalar and vector beams to be used in the same trap. As with traditional HOT, the hologram efficiencies must be adjusted to ensure the power delivered to each trap is as desired. In our experiments, we paid particular attention to this fact, particularly in the trapping stiffness tests, to ensure that only the polarization structures differed and that each beam has the same power at the trap. Related to this is the importance of due care with choice of optics in the delivery system in order to maintain the desired polarization structure of the beams in the vector HOT.

In this work, we have demonstrated the vector HOT using SLMs, HOPS beams and 2D arrays of traps. The SLM may be replaced with equivalent technology for faster refresh rates, e.g., digital micro-mirror devices (DMDs). Similarly, it should be clear from Eq. 1 that HOPS are an example only and that any arbitrary vector beam can be created at each location in the HOT array. Moreover, by switching off one component of the vector field at the SLM, the well-known scalar holographic trap can be reproduced, and as we have shown, vector and scalar beams may be produced at the same time in the same trap array. Given the many applications of scalar holographic traps, it is reasonable to assume that this new vector holographic trap too will find many applications, for example, in the trapping of gold nano-particles for plasmonic studies where vector beams have known benefits, inducing optical chirality in form birefringent structures, exploiting known vector trapping efficiency to adjust trapping strengths, e.g., in biological matter control with the same modal conditions (power and intensity profile) but differing polarizations for differing trap stengths, to execute fibre traps with vector modes, the natural modes of most fibres, and even in fundamental studies of light-matter interaction to compare phase singularities with polarization singularities (scalar OAM being an example of a phase singularity with the vector HOPS having a polarization singularity). Finally, by following the advances of scalar HOT, it will be possible to adjust the trapping plane of each vector beam by adding individual digital lenses to each beam^23^ for the realization of 3D traps^66^. We believe that since our system can produce both vector and scalar beams holographically at the same time, the addition of the polarization degree of freedom through vector beams will open new applications to follow.

To summarize, we have introduced and demonstrated the concept of a vector HOT for the delivery of 2D arrays of traps using HOPS beams. We created example arrays (square, triangular and linear) with combinations of scalar and vector HOPS beams, characterized the beams externally to the trap, and then demonstrated successful trapping with them. Each HOPS beam in the array was independently controlled by a SLM and spanned a wide range of positions on HOPS, including those associated with conventional scalar HOTs. By this approach, we were able to tailor on-demand the 3D shape of the optical forces at specific locations inside the optical trap, enabling the simultaneous realization of many traps with different trapping strength for the same laser power. We have pointed out how our vector HOT technique may be further advanced, which we hope will stimulate interest from the structured light and optical manipulation communities alike.

## Methods

### A vector HOT with multiple HOPS beams

**Figure 5:**
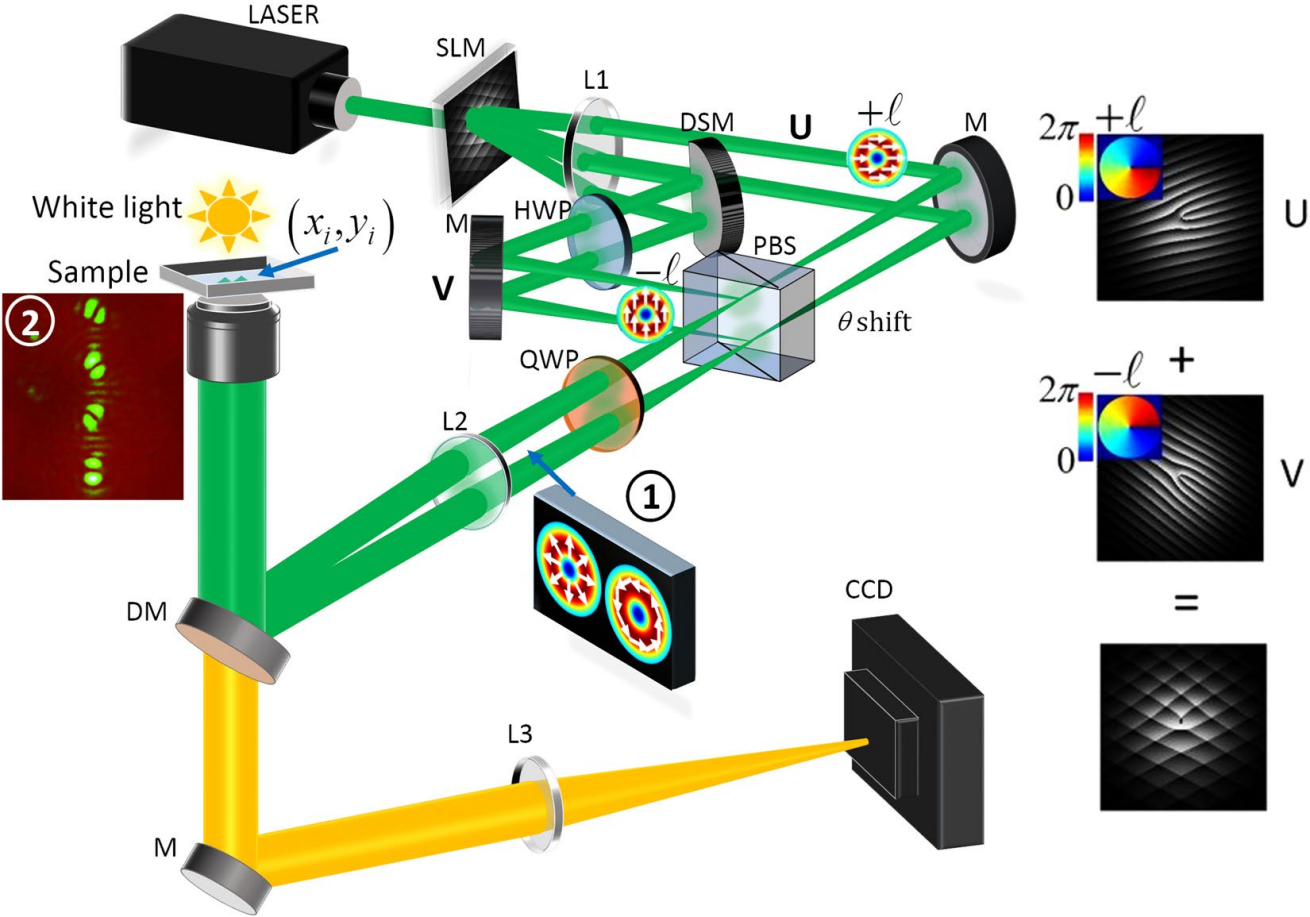
Schematic illustration of the experimental set-up used to simultaneously create multiple HOPS beam optical traps. An example multiplexed hologram pair used to generate two scalar beams traversing along two separate paths is shown in the insert on the right. Insert 1 shows example HOPS beams with radial and azimuthal polarization distributions. The generated vector beams were simultaneously directed into the microscope objective to create multiple optical traps. An inverted microscope comprising white light source, dichroic mirror, lens and CCD camera was used to image the trapped beads. Insert 2 shows an experimental image of a vertical array of vector beams in the trapping plane as observed on the CCD through back reflection off the sample container.

A schematic representation of the experimental setup used to generate arrays of optical traps with multiple vector beams is illustrated in Fig. 5. In our implementation, we used a horizontally polarized laser beam (*λ* = 532 nm, Coherent Verdi G) incident on a reflective SLM (Holoeye Pluto) aligned to modulate horizontal polarization for multiple vector beams generation. The inclusion of a telescope (lenses L1 and L2 in the figure; *f*1 = 500 mm, *f*2 = 1000 mm) is a crucial step required to send the HOPS beam into the optical trap. On one hand, by placing the lenses following a 4 f configuration, it images the SLM plane at the front focal plane of the microscope objective (100× Nikon oil immersion, N100X-PFO, NA 1.3). By doing this, unique propagation angles (as programmed on the SLM) will translate into unique (*x*_*i*_, *y*_*i*_) positions in the trapping plane: beams from the SLM propagate at differing angles which are mapped to positions with L1. The set-up converges the light at the PBS for interferometric recombination to produce the desired vector beams. To recreate the position control in the trap, L2 maps position to angle such that each vector beam incident at the objective will be focused to a unique position. On the other hand, focal length of the lenses of the telescope where chosen to ensure a 2*x* magnification to expand the beams and fill the entrance pupil of the microscope objective, ensuring tight focusing, then providing the necessary intensity gradient for trapping. A half wave plate (HWP) was introduced along path **V** to rotate the polarization from horizontal to vertical such that coaxial superposition of orthogonal polarization states was attained. The introduction of a HWP results in an optical path difference and potentially undesired phase delays which may cause a rotation in the polarization states of the generated vector beams. Since our created vector beams were cylindrical symmetric, the rotational effect cannot be observed. Nevertheless, to keep the system general, the optical path difference was corrected for by performing translation adjustments of the mirror in path **U**, countering the unwanted phase shift. The objective focused the beams into a sample container of depth 400 *μ*m holding 2 *μ*m diameter silica beads diluted in deionized water. An inverted microscope setup with an illuminating white light-emitting diode and a CCD camera was used to image the trapped beads. A dichroic mirror (DM) was used to reflect the vector beams into the objective while allowing white light from the light emitting diode to pass through to the CCD camera.

## Electronic supplementary material

Supplementary Information

## Data Availability

All data regarding the work presented here is available upon reasonable request to the corresponding author.
